# Sex-specific factors associated with lifetime suicide attempt among patients with alcohol use disorders

**DOI:** 10.1192/bjo.2022.545

**Published:** 2022-07-18

**Authors:** Susmita Pandey, Ingeborg Bolstad, Lars Lien, Fredrik A. Walby, Martin Øverlien Myhre, Jørgen G. Bramness

**Affiliations:** Norwegian National Advisory Unit on Concurrent Substance Abuse and Mental Health Disorders, Innlandet Hospital Trust, Hamar, Norway; and Institute of Clinical Medicine, University of Oslo, Oslo, Norway; Norwegian National Advisory Unit on Concurrent Substance Abuse and Mental Health Disorders, Innlandet Hospital Trust, Hamar, Norway; Norwegian National Advisory Unit on Concurrent Substance Abuse and Mental Health Disorders, Innlandet Hospital Trust, Hamar, Norway; and Department of Health and Social Science, Innlandet University of Applied Science, Elverum, Norway; National Centre for Suicide Research and Prevention, Institute of Clinical Medicine, University of Oslo, Oslo, Norway; National Centre for Suicide Research and Prevention, Institute of Clinical Medicine, University of Oslo, Oslo, Norway; Norwegian National Advisory Unit on Concurrent Substance Abuse and Mental Health Disorders, Innlandet Hospital Trust, Hamar, Norway; Department of Clinical Medicine, UiT – Norway's Arctic University, Tromsø, Norway; and Norwegian Institute of Public Health, Oslo, Norway

**Keywords:** Suicide attempt, alcohol use disorder, sex difference

## Abstract

**Background:**

Patients with alcohol use disorder (AUD) are at high risk for suicide attempts. Mental health problems along with AUD-related factors may contribute to this increased risk. Studies have shown sex differences in rates and correlates of suicide attempts.

**Aims:**

The purpose of the study was to examine mental-health-related and AUD-related factors associated with suicide attempt separately in female and male AUD patients.

**Method:**

We collected information about lifetime suicide attempt and mental-health- and AUD-related factors for AUD in-patients (*n* = 114; 32 females) receiving rehabilitative treatment.

**Results:**

The prevalence of lifetime suicide attempt was 27%, and the rate was similar in both sexes. Among females, current depressive symptoms and current post-traumatic stress disorder diagnosis were associated with suicide attempt. In male AUD patients, among the mental-health-related factors, lifetime major depression, panic disorder, social phobia, childhood sexual abuse and antisocial personality disorder were associated with suicide attempt. In addition, AUD-related factors including longer duration of drinking, history of delirium tremens, greater severity of AUD and lower levels of prolactin were associated with suicide attempt in males.

**Conclusions:**

Our results indicate that suicide attempts in female AUD patients were more mental-health-related, whereas those in males were also related to the severity of AUD. This suggests that a suicide prevention programme for AUD patients would benefit from a sex-based understanding of the risk factors.

Alcohol use disorder (AUD) is a potent risk factor for suicide.^1^ In fact, AUD is the second most important risk factor for suicide after major depression.^2^ Around 40% of AUD patients attempt suicide in their lifetime,^3,4^ and people who reattempt suicide are more likely to have AUD.^5^ Previous suicide attempt is among the strongest predictors of suicide,^6^ and the risk of suicide after suicide attempt may last for more than two decades.^7^ Therefore, a history of suicide attempt in AUD patients is clinically important.

## Factors related to suicide attempt among patients with AUD

Suicide attempt in AUD patients is related to demographic, mental-health-related and biological factors, in addition to AUD-related factors. Demographic factors including age, economic status and sex are related to suicide attempt among AUD patients.^4^ Mental-health-related factors such as mood disorders,^8^ anxiety disorders, post-traumatic stress disorder (PTSD),^9^ personality disorders including antisocial personality disorder (ASPD)^10^ and physical and sexual abuse^11^ are associated with suicide attempt in AUD patients. AUD-related factors such as early onset of alcohol use,^1^ chronicity of alcohol use,^12^ family history of AUD,^13^ recent problematic alcohol use^14^ and severity of AUD^11^ are also associated with suicide attempt. Furthermore, suicide attempt in AUD patients has been found to be related to neurobiological factors, including serotonin^15^ and dopamine^16^ dysregulation, which in turn may be attributed to alcohol use and are manifested, for instance, as a derangement in levels of circulating prolactin.^17^ Dopamine and serotonin exert regulatory functions over level of prolactins through inhibitory and stimulatory mechanisms, respectively.

## Possible sex differences in factors related to suicidal behaviour, mainly suicide attempt among patients with AUD

AUD patients are heterogenous and may be better understood when studied in subgroups. In that respect, sex-based differences have been evident from the very beginning in subtype-focused studies among AUD patients.^18^ Psychiatric comorbidities have been found to affect the AUD-based risk of suicide differently in females and males.^1^ Therefore, when studying suicide attempt in AUD patients, a sex-based understanding of the rate and risk factors is crucial. The literature is divided concerning differences in rates of suicide attempt between female and male AUD patients. Some report higher rates of suicide attempt in females^19,20^ and some in males,^21^ whereas others report no sex-based difference.^3,11^ Many studies on AUD patients have focused on sex as a covariate in predicting suicide attempt,^19^ but very few have explored the sex-specific predictors of suicide attempt.

One study in the general population reported that the repetition of suicide attempt was related to PTSD and depression severity in females and substance misuse in males.^22^ Another study in military veterans receiving treatment for substance misuse found that suicidal ideation and suicide attempts in females were more strongly related to the extent of misuse of alcohol and other substances, as well as to aggression and combat-related PTSD, whereas those in males were more strongly associated with sexual and physical abuse, depression and relationship problems.^23^ The sex differences reported by these studies, which moreover are inconsistent across different population groups, intensify the need to explore sex differences in AUD patients given the strong association of suicide attempt with AUD. Studies conducted among AUD patients found that in females, suicidal ideation was associated with comorbid psychopathology,^24^ including depression, childhood physical and sexual abuse, higher levels of aggression, and intensity and frequency of drinking.^25^ In male AUD patients, suicide attempt was associated with high impulsivity^26^ and ASPD, and with intensity but not frequency of drinking.^25^ Moreover, the risk of suicidal behaviour tends to increase with increasing AUD severity, and this effect is more prominent among females than males.^27^

## Study objectives

Prior studies have focused on the sex-specific associations of suicide with recently occurring AUD-related factors. However, suicide attempt in general may be attributed not solely to recent events but to a cumulative effect of events over time.^7^ To that end, there is a need to focus on the sex-specific associations of suicide with more enduring AUD-related historical parameters, such as duration of drinking. In this study, we aimed to assess sex-specific mental-health- and AUD-related factors, including enduring factors, that are associated with suicide attempt in AUD patients. We hypothesised that suicide attempt in female and male AUD patients would be associated with different mental-health- and AUD-related factors.

## Method

### Study participants

AUD patients (*n* = 114; 32 females) receiving in-patient treatment from three different rehabilitation clinics in Norway participated in the study. The median (25th, 75th percentiles) age of the participants was 53.4 (45.0, 57.9) years. The participants had been in treatment for a median (25th, 75th percentiles) of 7 days (5, 12) and had been abstinent for 19 (13, 28) days at the time of enrolment. The Norwegian Regional Ethics Committee (South-East B) provided ethical approval to conduct the study (reference number 2017/1314). The study was conducted in line with the Helsinki Declaration of 1975, as revised in 2008. Written informed consent was obtained from the patients before enrolment into the study. The data collection period for the study was January 2018 to August 2019.

Adults aged 18 years and over currently receiving in-patient treatment for AUD were included in the study. Patients who were unfamiliar with Scandinavian language or who were suffering from a severe somatic illness, psychosis or cognitive impairment that could limit their ability to provide informed consent or safely participate in the study were not included in the study.

### Measures

#### Patient characteristics

Information on the patient's age, sex, income and marital status were obtained.

#### Mental-health-related measures

Presence of lifetime suicide attempt was identified by asking the participants whether they had ever attempted suicide. The Beck Depression Inventory (BDI-II), a 21-item self-report inventory, was used to identify the severity of depression in the past 2 weeks.^28^ The study used the Norwegian validated version of BDI-II, for which Cronbach's alpha values ranging from 0.84 to 0.92 have been reported.^29^ Each item of the inventory consists of four statements which required self-evaluation, and the responses are scored from 0 to 3. The total score is the sum of individual responses and ranges from 0 to 63. Higher scores represent greater depression severity. In the present study, the Cronbach's alpha for BDI-II was 0.92.

History of trauma was assessed using a structured self-report questionnaire which has been used previously to interview a psychiatric population.^30^ The questionnaire consisted of five questions, where the first three were related to childhood trauma and the last two were related to adult trauma. These questions asked about sexual abuse, physical abuse and any other traumatic events. A positive response on any of the first three questions was considered to indicate the presence of childhood trauma, and a positive response on either of the last two questions was considered to indicate the presence of adulthood trauma.

The Mini International Neuropsychiatric Interview (M.I.N.I.) Norwegian translation version 6.0 was used to screen for lifetime major depression, panic disorder, ASPD, current social phobia, and PTSD. M.I.N.I. 6.0 is a short structured diagnostic interview for DSM-IV psychiatric disorders, and the translated version has demonstrated acceptable psychometric properties.^31^

#### AUD-related measures

##### Self-report measures

Information on duration of drinking (years), alcohol problems in parents, history of delirium tremens and previous AUD treatment was obtained. The severity of AUD was identified using the Severity of Dependence Scale (SDS), which measures impaired control over drug-taking, preoccupation and anxiety regarding drug use over the past year.^32^ We used the Norwegian version of the SDS, for which Cronbach's alpha values ranging from 0.72 to 0.80 across a variety of substances have been reported.^33,34^ The instrument consists of five items, and each item is scored on a four-point Likert scale (0 to 3). The individual scores are summed, and higher scores represent more severe AUD. In this study, the internal consistency of SDS as measured by Cronbach's alpha was 0.78.

##### Biological measures

Levels of phosphatidylethanol (PEth; 16:0/18:1) and serum prolactin were examined. PEth was assessed twice, once at baseline and then at 6 week follow-up. PEth was measured by supercritical fluid chromatography mass spectrometry and prolactin was measured by chemiluminescence immunoassay.

### Missing data

Some variables, including age, sex, social phobia and PTSD, had no missing data. When variables had missing data at a unit level, the person with missing data was removed from the analyses. Those variables were income, marital status, lifetime major depression, panic disorder, trauma, ASPD and all AUD-related measures. Variable-wise sample sizes used in the analysis are presented in Table 1. BDI-II had missing data at an item level; therefore, person–mean imputation was done when 17 or more of the 21 items were responded to.

**Table 1:** Differences between alcohol use disorder (AUD) patients with and without lifetime suicide attempts (*n* = 114)

Factors with possible influence on suicide attempt		Lifetime suicide attempt				
		Absent *n* = 83 (73%)		Present *n* = 31(27%)		*P*-value
		*n*		*n*		
**Patient characteristics**						
Age (years)	Median (25th, 75th percentile)	83	53.3 (45.2, 58.2)	31	53.9 (44.2, 57.8)	0.916^a^
Sex (female)	*n* (%)	83	21 (25.3)	31	11 (35.5)	0.282^b^
Having paid job as a source of income	*n* (%)	64	15 (23.4)	24	2 (8.3)	0.138^b^
Marital status (having a partner)	*n* (%)	65	16 (24.6)	24	6 (25.0)	0.970^b^
**Mental-health-related measures**						
Current depressive score (BDI-II)	Median (25th, 75th percentile)	61	14.0 (8.0, 22.0)	24	25.0 (12.0, 30.8)	**0**.**016**^a^
Had lifetime major depression	*n* (%)	82	56 (68.3)	31	27 (87.1)	**0**.**043**^b^
Panic disorder lifetime	*n* (%)	82	32 (39.0)	31	21 (67.7)	**0**.**006**^b^
Social phobia	*n* (%)	83	13 (15.7)	31	10 (32.3)	**0**.**049**^b^
Presence of childhood sexual abuse	*n* (%)	62	9 (14.5)	24	10 (41.7)	**0**.**006**^b^
Presence of childhood trauma	*n* (%)	62	45 (72.6)	24	16 (66.7)	0.588^b^
Presence of adult trauma	*n* (%)	62	36 (58.1)	24	18 (75.0)	0.145^b^
Having post-traumatic stress disorder	*n* (%)	83	10 (12.0)	31	10 (32.3)	**0**.**012**^b^
Having antisocial personality disorder	*n* (%)	83	9 (10.8)	30	8 (26.7)	0.069^c^
**AUD-related factors**						
***Self-report measures***						
Duration of drinking (years)	Median (25th, 75th percentile)	82	12.5 (6.8, 20.3)	31	18.0 (10.0, 30.0)	**0**.**046**^a^
Parents having drinking problem	*n* (%)	82	45 (54.9)	31	16 (51.6)	0.756
Severity of dependence scale	Median (25th, 75th percentile)	63	10.0 (7.0,11.0)	24	11.0 (9.3, 13.0)	**0**.**007**^a^
History of delirium tremens	*n* (%)	82	15 (18.3)	31	12 (38.7)	**0**.**023**^b^
History of previous AUD treatment	*n* (%)	82	51 (62.2)	31	23 (74.2)	0.231
***Biological measures***						
Level of PEth (μmol/L)	Median (25th, 75th percentile)	78	0.3 (0.1, 0.7)	31	0.2 (0.1, 0.6)	0.527^a^
Level of prolactin (mU/L)	Median (25th, 75th percentile)	82	136.0 (107.5, 196.5)	30	126.0 (85.8, 161.5)	0.227^a^

BDI, Beck Depression Inventory; PEth, phosphatidylethanol.

a. Mann–Whitney's U-test.

b. Chi-squared test.

c. Fisher's exact test.

### Statistical analyses

SPSS version 23.0 for Windows was used to perform the statistical analyses. Characteristics of AUD patients with and without suicide attempt were presented using descriptive statistics. The values were not normally distributed, as indicated by Shapiro–Wilk test, and are therefore presented as medians and 25th and 75th percentiles in the tables. For comparisons between groups, chi-squared or Fisher's exact test was used for categorical variables, and Mann–Whitney *U*-test was used for continuous variables. Furthermore, logistic regression was performed to identify sex-specific factors associated with suicide attempt and odds ratios (OR) with 95% confidence intervals were reported. The variables that elicited a *P*-value of less than 0.150 on groupwise comparison for each gender were included in the logistic regression model for the respective gender. In addition, for mental-health-related variables, an adjusted logistic regression model was built by adjusting for age and duration of drinking. Similarly, for AUD-related variables, a model adjusting for age and lifetime major depression was built. For male AUD patients, an additional model was built for mental-health-related variables, adjusting for age and lifetime major depression. Statistical tests were two-tailed with a significance level of *α* = 0.05.

## Results

The prevalence of suicide attempt in AUD in-patients was 27%. Table 1 shows the differences between AUD patients with and without suicide attempt. There was no sex difference in the prevalence of suicide attempt. Those who had attempted suicide had higher current depressive scores, and they more frequently met the criteria for lifetime major depression, lifetime panic disorder, social phobia and PTSD. In addition, they more frequently reported experiencing sexual abuse as a child. Moreover, the patients who had attempted suicide had a longer duration of drinking, had more frequently reported a history of delirium tremens and had more severe AUD compared with those who did not attempt suicide.

Higher current depressive symptoms and presence of PTSD were associated with suicide attempt among females (Table 2). In the unadjusted logistic regression analysis (Table 4), the OR for current depressive symptoms was 1.14 (95% CI: 1.02, 1.28) and that for PTSD was 7.92 (95% CI: 1.21, 51.84). After adjustment for age and duration of drinking, the association between current depressive score and suicide attempt was no longer statistically significant, but the association of suicide attempt with the presence of PTSD remained significant (OR = 8.82, 95% CI: 1.12, 69.54).

**Table 2:** Factors associated with lifetime suicide attempts in female alcohol use disorder (AUD) patients (*n* = 32)

Factors with possible influence on suicide attempt		Lifetime suicide attempt in female AUD patients				
		Absent *n* = 21 (66%)		Present *n* = 11 (34%)		*P*-value
		*N*		*N*		
**Patient characteristics**						
Age (years)	Median (25th, 75th percentile)	21	51.5 (42.9, 55.3)	11	53.0 (44.2, 57.8)	0.827^a^
Having paid job as a source of income	*n* (%)	15	4 (26.7)	7	0 (0.0)	0.263^b^
Marital status (having a partner)	*n* (%)	16	5 (31.3)	7	2 (28.6)	1.000^b^
**Mental-health-related measures**						
Current depressive score (BDI-II)	Median (25th, 75th percentile)	15	19.0 (12.0, 25.0)	7	32.0 (27.0, 44.0)	**0**.**010**^a^
Had lifetime major depression	*n* (%)	21	19 (90.5)	11	10 (90.9)	1.000^b^
Panic disorder lifetime	*n* (%)	21	13 (61.9)	11	9 (81.8)	0.425^b^
Social phobia	*n* (%)	21	3 (14.3)	11	3 (27.3)	0.390^b^
Presence of childhood sexual abuse	*n* (%)	15	5 (33.3)	7	5 (71.4)	0.172^b^
Presence of childhood trauma	*n* (%)	15	15 (100.0)	7	7 (100.0)	
Presence of adult trauma	*n* (%)	15	15 (100.0)	7	7 (100.0)	
Having post-traumatic stress disorder	*n* (%)	21	2 (9.5)	11	5 (45.5)	**0**.**032**^b^
Having antisocial personality disorder	*n* (%)	21	0 (0.0)	10	1 (10.0)	0.323^b^
**AUD-related factors**						
***Self-report measures***						
Duration of drinking (years)	Median (25th, 75th percentile)	21	7.0 (5.5, 15.0)	11	11.0 (2.0, 18.0)	0.426^a^
Parents having drinking problem	*n* (%)	21	13 (61.9)	11	6 (54.5)	0.721^b^
History of delirium tremens	*n* (%)	21	1 (4.8)	11	2 (18.2)	0.266^b^
History of previous AUD treatment	*n* (%)	21	10 (47.6)	11	7 (63.6)	0.388^c^
Severity of dependence scale	Median (25th, 75th percentile)	15	10.0 (10.0, 13.0)	7	11.0 (9.0, 14.0)	0.334^a^
***Biological measures***						
Level of PEth (μmol/L)	Median (25th, 75th percentile)	18	0.3 (0.1, 0.7)	11	0.2 (0.0, 0.4)	0.200^a^
Level of prolactin (mU/L)	Median (25th, 75th percentile)	21	125.0 (109.0, 196.0)	11	159.0 (103.0, 242.0)	0.781^a^

BDI, Beck Depression Inventory; PEth, phosphatidylethanol.

a. Mann–Whitney U-test.

b. Fisher's exact test.

c. Chi-squared test.

Suicide attempt in males (Table 3) was associated with the presence of lifetime major depression, lifetime panic disorder, childhood sexual abuse, longer duration of drinking, history of delirium tremens and more severe AUD.

**Table 3:** Factors associated with lifetime suicide attempts in male alcohol use disorder (AUD) patients (*n* = 82)

Factors with possible influence on suicide attempt		Lifetime suicide attempt in male AUD patients				
		Absent *n* = 62 (76%)		Present *n* = 20 (24%)		*P*-value
		*n*		*n*		
**Patient characteristics**						
Age (years)	Median (25th, 75th percentile)	62	53.5 (45.2, 60.0)	20	53.9 (44.1, 60.6)	0.871^a^
Having paid job as a source of income	*n* (%)	49	11 (22.4)	17	2 (11.8)	0.488^b^
Marital status (having a partner)	*n* (%)	49	11 (22.4)	17	4 (23.5)	1.000^b^
**Mental-health-related measures**						
Current depressive score (BDI-II)	Median (25th, 75th percentile)	46	13.0 (7.8, 21.0)	17	15.0 (11.0, 27.5)	0.156^a^
Had lifetime major depression	*n* (%)	61	37 (60.7)	20	17 (85.0)	**0**.**045**^c^
Panic disorder lifetime	*n* (%)	61	19 (31.1)	20	12 (60.0)	**0**.**021**^c^
Social phobia	*n* (%)	62	10 (16.1)	20	7 (35.0)	0.110^b^
Presence of childhood sexual abuse	*n* (%)	47	4 (8.5)	17	5 (29.4)	**0**.**048**^b^
Presence childhood trauma	*n* (%)	47	30 (63.8)	17	9 (52.9)	0.430^c^
Presence of adult trauma	*n* (%)	47	21 (44.7)	17	11 (64.7)	0.157^c^
Having post-traumatic stress disorder	*n* (%)	62	8 (12.9)	20	5 (25.0)	0.288^b^
Having antisocial personality disorder	*n* (%)	62	9 (14.5)	20	7 (35.0)	0.057^b^
**AUD-related factors**						
***Self-report measures***						
Duration of drinking (years)	Median (25th, 75th percentile)	61	16.0 (7.5, 22.0)	20	22.5 (15.0, 30.0)	**0**.**025**^a^
Parents having drinking problem	*n* (%)	61	32 (52.5)	20	10 (50.0)	0.849^c^
History of delirium tremens	*n* (%)	61	14 (23.0)	20	10 (50.0)	**0**.**022**^c^
History of previous AUD treatment	*n* (%)	61	41 (67.2)	20	16 (80.0)	0.277^c^
Severity of dependence scale	Median (25th, 75th percentile)	48	9.5 (6.3, 11.0)	17	11.0 (9.5, 12.5)	**0**.**005**^a^
***Biological measures***						
Level of PEth (μmol/L)	Median (25th, 75th percentile)	60	0.3 (0.1, 0.6)	20	0.3 (0.1, 0.6)	0.902^a^
Level of prolactin (mU/L)	Median (25th, 75th percentile)	61	138.0 (105.5, 197.5)	19	124.0 (72.0, 156.0)	0.078^a^

BDI, Beck Depression Inventory; PEth, phosphatidylethanol.

a. Mann–Whitney U-test.

b. Fisher's exact test.

c. Chi-squared test.

**Table 4:** Logistic regression model of lifetime suicide attempt among female patients with alcohol use disorder (*n* = 32)

	Reference		Unadjusted	*P*-value	Adjusted for age and duration of drinking	*P*-value
**Patient characteristic**						
Age	Continuous	OR (95% CI)	1.00 (0.92−1.07)	0.892		
**Mental-health-related measures**						
Current depressive score (BDI-II)	Continuous	OR (95% CI)	1.14 (1.02−1.28)	**0**.**025**	1.14 (1.00−1.30)	0.056
Having PTSD	Not having PTSD	OR (95% CI)	7.92 (1.21−51.84)	**0**.**031**	8.82 (1.12−69.54)	**0**.**039**

OR, odds ratio; CI, confidence interval; BDI, Beck Depression Inventory; PTSD, post-traumatic stress disorder.

Table 5 shows the unadjusted and adjusted logistic regression models for suicide attempt in males. In the unadjusted analysis, presence of lifetime panic disorder (OR = 3.32, 95% CI: 1.17, 9.44), experiencing childhood sexual abuse (OR = 4.48, 95% CI: 1.04, 19.33), longer duration of drinking (OR = 1.05, 95% CI: 1.00, 1.11), history of delirium tremens (OR = 3.36, 95% CI: 1.16, 9.69) and more severe AUD (OR = 1.39, 95% CI: 1.07, 1.80) were associated with suicide attempt, but presence of lifetime major depression was not.

**Table 5:** Logistic regression model of lifetime suicide attempt among male patients with alcohol use disorder (AUD) (*n* = 82)

	Reference		Unadjusted	*P*-value	Model 1^a^	*P*-values	Model 2^a^	*P*-value
					Adjusted for lifetime major depression and age		Adjusted for duration of drinking and age	
**Patient characteristic**								
Age	Continuous	OR (95% CI)	1.00 (0.96−1.05)	0.897				
**Mental-health-related measures**								
Had lifetime major depression	Never had	OR (95% CI)	3.68 (0.97−13.91)	0.055			4.27 (1.05−17.47)	**0**.**043**
Panic disorder lifetime	Absence	OR (95% CI)	3.32 (1.17−9.44)	0.025	2.79 (0.91−8.58)	0.074	3.05 (0.99−9.41)	0.053
Social phobia	Absence	OR (95% CI)	2.80 (0.89−8.77)	0.077	2.02 (0.59−6.91)	0.265	4.17 (1.16−15.02)	**0**.**029**
Presence of childhood sexual abuse	Absence	OR (95% CI)	4.48 (1.04−19.33)	0.044	4.62 (1.07−19.93)	**0**.**040**	4.88 (1.14−20.89)	**0**.**033**
Having antisocial personality disorder	Not having	OR (95% CI)	3.17 (1.00−10.10)	0.051	5.72 (1.30−25.16)	**0**.**021**	6.69 (1.50−29.76)	**0**.**013**
**AUD-related factors**								
***Self-report measures***								
Duration of drinking (years)	Continuous	OR (95% CI)	1.05 (1.00−1.11)	0.031	1.06 (1.01−1.12)	**0**.**029**		
History of delirium tremens	No history	OR (95% CI)	3.36 (1.16−9.69)	0.025	2.83 (0.95−8.41)	0.061		
Severity of dependence scale	Continuous	OR (95% CI)	1.39 (1.07−1.80)	0.013	1.38 (1.05−1.81)	**0**.**020**		
***Biological measures***								
Level of prolactin (mU/L)	Continuous	OR (95% CI)	0.99 (0.98−1.00)	0.056	0.99 (0.98−1.00)	**0**.**042**		

OR, odds ratio; CI, confidence interval.

^a^Model 1 was adjusted for age and lifetime major depression. Model 2 was adjusted for age and duration of drinking.

Two logistic regression models were built for the mental-health-related variables. The first model was adjusted for age and lifetime major depression; with this model, presence of childhood sexual abuse (OR = 4.62, 95% CI: 1.07, 19.93) and ASPD (OR = 5.72, 95% CI: 1.30, 25.16) were associated with suicide attempt. The second model was adjusted for age and duration of drinking; here, lifetime major depression (OR = 4.27, 95% CI: 1.05, 17.47), social phobia (OR = 4.17, 95% CI: 1.16, 15.02), childhood sexual abuse (OR = 4.88, 95% CI: 1.14, 20.89) and ASPD (OR = 6.69, 95% CI: 1.50, 29.76) were associated with suicide attempt. In a *post hoc* analysis, where we included childhood sexual abuse and ASPD in the same model, we found that childhood sexual abuse was still associated with suicide attempt (OR = 4.47, 95% CI: 1.00, 19.89; not shown in the table) but ASPD was not associated (OR = 2.84, 95% CI: 0.70, 11.55; not shown in the table).

For the AUD-related variables, a model adjusted for age and lifetime major depression was built; according to this model, duration of drinking (OR = 1.06, 95% CI: 1.01, 1.12), severity of AUD (OR = 1.38, 95% CI: 1.05, 1.81) and level of prolactin (OR = 0.99, 95% CI: 0.98, 1.00) were associated with suicide attempt.

## Discussion

### Main findings

This study of AUD in-patients identified a similar prevalence of suicide attempt in females and males, but factors associated with suicide attempt differed based on sex. In females, higher levels of current depressive symptoms and presence of PTSD were associated with suicide attempt. In males, mental-health-related variables including lifetime major depression, panic disorder, social phobia, childhood sexual abuse and ASPD were associated with suicide attempt. In addition, AUD-related variables including longer duration of drinking, history of delirium tremens, more severe AUD and lower levels of serum prolactin were associated with suicide attempt in male AUD patients.

### Interpretation of the findings

In line with our findings, some studies have reported no sex difference in the rate of suicide attempt,^3,11^ whereas others report higher rates in either female^19,20^ or male^21^ AUD patients. Some of these studies examined lifetime suicide attempt, whereas others studied recent suicide attempt or suicide attempt during a specific follow-up period; however, in any case, findings on the predictive role of sex in suicide attempt in AUD patients are not consistent. On the other hand, in the general population and most clinical samples, studies tend to converge towards the finding of higher suicidal behaviour among females.^35^

Depression is a strong risk factor for suicide.^2^ In our study, among females, lifetime suicide attempt was associated with higher levels of current depressive symptoms but not lifetime major depression. This could have been because more than 90% of the females in the study met the criteria for lifetime major depression, leading to a possible ceiling effect. Nevertheless, lifetime suicide attempt was associated with the presence of lifetime major depression among males. The presence of current depressive symptoms may represent a depressive disorder with more frequent episodes or more enduring symptoms and could therefore distinguish patients with a more severe depressive disorder from those with lifetime major depression. The association of current depressive symptoms with suicide attempt only in females may indicate that females who attempt suicide have more severe depressive disorders than males, and that depression-related diathesis to suicide attempt could therefore be higher in females. Association of comorbid PTSD with suicide attempt in AUD patients has been reported previously.^36^ Our finding of the association between PTSD and suicide attempt only in females suggests that the presence of PTSD might be riskier in females than in males in terms of suicide attempt.

Among males, panic disorder and social phobia were associated with suicide attempt. This has been reported previously in AUD patients^37^ and in a sex-unstratified general population.^8^ Furthermore, we found that childhood sexual abuse was associated with suicide attempt in males; this was also reported by another study in AUD patients, although that study population was not stratified by sex.^11^ Reports from the literature suggest that childhood sexual abuse is a risk factor for AUD as well as for suicidal behaviour.^38^ In our study, all females had experienced childhood and adult trauma; thus, its effect on suicide attempt could not be detected. In addition, we found that the presence of ASPD in males was associated with suicide attempt. This was consistent with the findings of a study of male veterans with AUD.^39^ Furthermore, impulsivity and aggression, which are key features of ASPD, have been reported as important risk factors for suicidal behaviour in AUD patients.^40^ The association of ASPD with suicide attempt in males persisted even after adjusting for childhood sexual abuse. In female AUD patients, only one patient had ASPD; therefore, our study was underpowered to find an association of ASPD with suicide attempt.

Among the AUD-related variables, we found that a longer duration of drinking was related to suicide attempt in males, consistent with the findings of studies that examined suicide risk^41^ and suicide ideation^12^ in sex-unstratified samples. We propose two possible explanations for the association of longer duration of drinking with suicide attempt. First, a longer duration of drinking may reflect a longer duration of mental-health-related problems that are associated with AUD but also with suicide. Second, a longer duration of drinking might have eventuated neurobiological alterations, such as dopaminergic and serotonergic dysfunction, which in turn potentiate suicidal behaviour.^42^ Furthermore, studies have reported an association of AUD-related variables with suicidal ideas and attempts in females,^23,25^ but we did not find this in our study.

Among the males, we also found an association of a history of delirium tremens with suicide attempt. This is consistent with findings from studies not stratified by sex.^43^ However, many case studies that have reported self-mutilation associated with delirium tremens were based on males.^44,45^ In our study, only three of the female participants had a history of delirium tremens, which was probably not enough to detect the influence of delirium tremens in females. Strengthening our finding of the association of a history of delirium tremens with suicide attempt, we also found that suicide attempt was associated with greater AUD severity in male AUD patients. Many previous studies report similar findings, even if they did not stratify by sex.^3,11^

In addition, a lower level of prolactin was associated with suicide attempt in male AUD patients. An earlier study demonstrated that lower levels of prolactin could predicted suicide attempt in female psychiatric patients.^46^ As mentioned earlier, levels of prolactin may be related to dopamine and serotonin, with the former having an inhibitory effect and the latter a stimulatory effect on prolactin release.^17^ It has been proposed that a combination of low serotonin and high dopamine function serves as a neurobiological trait in predisposing impulsive aggression, and this in turn is associated with suicidality.^47^ Moreover, impulsive aggression is characteristic of ASPD, and in our study ASPD was also related to suicide attempt. Collectively, our finding of lower prolactin and the presence of ASPD being associated with suicide attempt only in male AUD patients may indicate that males had made more impulsive suicide attempts than female AUD patients.

### Limitations and implications

The major strength of this study was the comprehensiveness of covariates from several domains for investigating the sex-based associations of suicide attempt. However, especially among females, the study suffered from underpowering as well as ceiling effects for some variables, increasing its vulnerability towards type II statistical errors. In addition, we observed wide 95% confidence intervals for some of the variables, such as PTSD among females, and childhood sexual abuse and ASPD among males, reflecting a greater degree of uncertainty of the estimates, possibly owing to the low sample sizes for these variables. Moreover, some variables relevant to suicide attempt, such as frequency and recency of suicide attempt, presence of borderline personality disorder, family history of suicide, interpersonal stress and negative life events, were not investigated in the current study. Another limitation of the study was the lack of distinction between proximal and distal risk factors for suicide attempt. Our study suggests that suicide prevention interventions among AUD patients would benefit from a sex-based understanding of the risk factors. Furthermore, whereas the existing literature on suicide attempt among AUD patients focuses more on alcohol intoxication and current withdrawal symptoms as risk factors, our findings suggest that future studies need to focus additionally on more enduring AUD-related factors. Our preliminary findings on sex-specific factors associated with suicide attempt in AUD patients need to be replicated by larger studies, especially by those that are adequately powered for female AUD patients.

In conclusion, the prevalence of suicide attempt in female and male AUD patients was similar, but the factors associated with suicide attempt were different. Whereas mental-health-related factors were associated with suicide attempt in both sexes, AUD-related factors were related to suicide attempt only in males. In addition, there were differences within the mental-health-related factors associated with suicide attempt in males and females. More often, lifetime mental-health-related factors were significant for males, whereas only current mental-health-related factors were significant for females. Association of suicide attempt with current depressive symptoms and PTSD in female AUD patients may indicate the presence of a more frequent or long-lasting psychopathology in females contributing to their diathesis for suicide attempt.

## Data Availability

The data are available from the corresponding author (S.P.) upon reasonable request.

## References

[1] Edwards AC, Ohlsson H, Sundquist J, Sundquist K, Kendler KS. Alcohol use disorder and risk of suicide in a Swedish population-based cohort. Am J Psychiatry 2020; 177(7): 627–34. 32160767 10.1176/appi.ajp.2019.19070673 PMC8887810

[2] Ferrari AJ, Norman RE, Freedman G, Baxter AJ, Pirkis JE, Harris MG, The burden attributable to mental and substance use disorders as risk factors for suicide: findings from the Global Burden of Disease Study 2010. PLoS One 2014; 9(4): e91936. 24694747 10.1371/journal.pone.0091936 PMC3973668

[3] Wojnar M, Ilgen MA, Czyz E, Strobbe S, Klimkiewicz A, Jakubczyk A, Impulsive and non-impulsive suicide attempts in patients treated for alcohol dependence. J Affect Disord 2009; 115(1–2): 131–9. 18835498 10.1016/j.jad.2008.09.001 PMC2702673

[4] Sher L. Risk and protective factors for suicide in patients with alcoholism. ScientificWorldJournal 2006; 6: 1405–11. 17086346 10.1100/tsw.2006.254 PMC5917221

[5] Parra-Uribe I, Blasco-Fontecilla H, Garcia-Parés G, Martínez-Naval L, Valero-Coppin O, Cebrià-Meca A, Risk of re-attempts and suicide death after a suicide attempt: a survival analysis. BMC Psychiatry 2017; 17(1): 163. 28472923 10.1186/s12888-017-1317-z PMC5415954

[6] Bostwick JM, Pabbati C, Geske JR, McKean AJ. Suicide attempt as a risk factor for completed suicide: even more lethal than we knew. Am J Psychiatry 2016; 173(11): 1094–100. 27523496 10.1176/appi.ajp.2016.15070854 PMC5510596

[7] Probert-Lindström S, Berge J, Westrin Å, Öjehagen A, Skogman Pavulans K. Long-term risk factors for suicide in suicide attempters examined at a medical emergency in patient unit: results from a 32-year follow-up study. BMJ Open 2020; 10(10): e038794. 10.1136/bmjopen-2020-038794 PMC7783608 33130567

[8] Nepon J, Belik S-L, Bolton J, Sareen J. The relationship between anxiety disorders and suicide attempts: findings from the national epidemiologic survey on alcohol and related conditions. Depress Anxiety 2010; 27(9): 791–8. 20217852 10.1002/da.20674 PMC2940247

[9] Yuodelis-Flores C, Ries RK. Addiction and suicide: a review. Am J Addict 2015; 24(2): 98–104. 25644860 10.1111/ajad.12185

[10] Hesselbrock M, Hesselbrock V, Syzmanski K, Weidenman M. Suicide attempts and alcoholism. J Stud Alcohol 1988; 49(5): 436–42. 3216647 10.15288/jsa.1988.49.436

[11] Jakubczyk A, Klimkiewicz A, Krasowska A, Kopera M, Sławińska-Ceran A, Brower KJ, History of sexual abuse and suicide attempts in alcohol-dependent patients. Child Abuse Negl 2014; 38(9): 1560–8. 24997776 10.1016/j.chiabu.2014.06.010 PMC4601637

[12] Duncan SC, Alpert A, Duncan TE, Hops H. Adolescent alcohol use development and young adult outcomes. Drug Alcohol Depend 1997; 49(1): 39–48. 9476698 10.1016/s0376-8716(97)00137-3

[13] Sung Y-k, La Flair LN, Mojtabai R, Lee L-C, Spivak S, Crum RM. The association of alcohol use disorders with suicidal ideation and suicide attempts in a population-based sample with mood symptoms. Arch Suicide Res 2016; 20(2): 219–32. 25933091 10.1080/13811118.2015.1004489 PMC5728356

[14] Ilgen MA, Harris AHS, Moos RH, Tiet QQ. Predictors of a suicide attempt one year after entry into substance use disorder treatment. Alcohol Clin Exp Res 2007; 31(4): 635–42. 17374043 10.1111/j.1530-0277.2007.00348.x

[15] Pompili M, Serafini G, Innamorati M, Dominici G, Ferracuti S, Kotzalidis GD, Suicidal behavior and alcohol abuse. Int J Environ Res Public Health 2010; 7(4): 1392–431. 20617037 10.3390/ijerph7041392 PMC2872355

[16] Jasiewicz A, Samochowiec A, Samochowiec J, Małecka I, Suchanecka A, Grzywacz A. Suicidal behavior and haplotypes of the dopamine receptor gene (DRD2) and ANKK1 gene polymorphisms in patients with alcohol dependence–preliminary report. PLoS One 2014; 9(11): e111798. 25415204 10.1371/journal.pone.0111798 PMC4240548

[17] Markianos M, Hatzimanolis J, Lykouras L. Relationship between prolactin responses to ECT and dopaminergic and serotonergic responsivity in depressed patients. Eur Arch Psychiatry Clin Neurosci 2002; 252(4): 166–71. 12242577 10.1007/s00406-002-0377-2

[18] Cloninger CR, Sigvardsson S, Bohman M. Type I and type II alcoholism: an update. Alcohol Health Res World 1996; 20(1): 18–23. 31798167 PMC6876531

[19] Roy A, Janal MN. Risk factors for suicide attempts among alcohol dependent patients. Arch Suicide Res 2007; 11(2): 211–7. 17453699 10.1080/13811110701250150

[20] Curlee J. A comparison of male and female patients at an alcoholism treatment center. J Psychol 1970; 74(2): 239–47. 4391867 10.1080/00223980.1970.9923735

[21] Boenisch S, Bramesfeld A, Mergl R, Havers I, Althaus D, Lehfeld H, The role of alcohol use disorder and alcohol consumption in suicide attempts-a secondary analysis of 1921 suicide attempts. Eur Psychiatry 2010; 25(7): 414–20. 20627467 10.1016/j.eurpsy.2009.11.007

[22] Monnin J, Thiemard E, Vandel P, Nicolier M, Tio G, Courtet P, Sociodemographic and psychopathological risk factors in repeated suicide attempts: gender differences in a prospective study. J Affect Disord 2012; 136(1-2): 35–43. 21975134 10.1016/j.jad.2011.09.001

[23] Benda BB. Gender differences in predictors of suicidal thoughts and attempts among homeless veterans that abuse substances. Suicide Life Threat Behav 2005; 35(1): 106–16. 15843327 10.1521/suli.35.1.106.59262

[24] Agrawal A, Constantino AM, Bucholz KK, Glowinski A, Madden PAF, Heath AC, Characterizing alcohol use disorders and suicidal ideation in young women. J Stud Alcohol Drugs 2013; 74(3): 406–12. 23490569 10.15288/jsad.2013.74.406 PMC3602360

[25] Conner KR, Li Y, Meldrum S, Duberstein PR, Conwell Y. The role of drinking in suicidal ideation: analyses of Project MATCH data. J Stud Alcohol 2003; 64(3): 402–8. 12817830 10.15288/jsa.2003.64.402

[26] Bergman B, Brismar B. Hormone levels and personality traits in abusive and suicidal male alcoholics. Alcohol Clin Exp Res 1994; 18(2): 311–6. 8048732 10.1111/j.1530-0277.1994.tb00019.x

[27] Jeong J-E, Joo S-H, Hahn C, Kim D-J, Kim T-S. Gender-specific association between alcohol consumption and stress perception, depressed mood, and suicidal ideation: the 2010-2015 KNHANES. Psychiatry Invest 2019; 16(5): 386–96. 10.30773/pi.2019.02.28 PMC6539269 31132843

[28] Beck AT, Ward CH, Mendelson M, Mock J, Erbaugh J. An inventory for measuring depression. Arch Gen Psychiatry 1961; 4: 561–71. 13688369 10.1001/archpsyc.1961.01710120031004

[29] Siqveland J, Kornør H. Måleegenskaper ved den norske versjonen av Beck Depression Inventory (BDI II). PsykTestBarn 2011; 1(5).

[30] Toft H, Neupane SP, Bramness JG, Tilden T, Wampold BE, Lien L. The effect of trauma and alcohol on the relationship between level of cytokines and depression among patients entering psychiatric treatment. BMC Psychiatry 2018; 18(1): 95. 29631540 10.1186/s12888-018-1677-z PMC5891976

[31] Mordal J, Gundersen Ø, Bramness J. Norwegian version of the mini-international neuropsychiatric interview: feasibility, acceptability and test-retest reliability in an acute psychiatric ward. Eur Psychiatry 2010; 25(3): 172–7. 19553089 10.1016/j.eurpsy.2009.02.004

[32] Gossop M, Darke S, Griffiths P, Hando J, Powis B, Hall W, The severity of dependence scale (SDS): psychometric properties of the SDS in English and Australian samples of heroin, cocaine and amphetamine users. Addiction 1995; 90(5): 607–14. 7795497 10.1046/j.1360-0443.1995.9056072.x

[33] Cheng S, Siddiqui TG, Gossop M, Kristoffersen ES, Lundqvist C. The severity of dependence scale detects medication misuse and dependence among hospitalized older patients. BMC Geriatr 2019; 19(1): 174. 31234786 10.1186/s12877-019-1182-3 PMC6591833

[34] Kristoffersen ES, Benth J, Straand J, Russell MB, Lundqvist C. Validity of self-reported assessment of severity of dependence scale in medication-overuse headache. Scand J Pain 2019; 19(4): 837–41. 31199778 10.1515/sjpain-2019-0022

[35] Borges G, Nock MK, Haro Abad JM, Hwang I, Sampson NA, Alonso J, Twelve-month prevalence of and risk factors for suicide attempts in the world health organization world mental health surveys. J Clin Psychiatry 2010; 71(12): 1617–28. 20816034 10.4088/JCP.08m04967blu PMC3000886

[36] Rojas SM, Bujarski S, Babson KA, Dutton CE, Feldner MT. Understanding PTSD comorbidity and suicidal behavior: associations among histories of alcohol dependence, major depressive disorder, and suicidal ideation and attempts. J Anxiety Disord 2014; 28(3): 318–25. 24681282 10.1016/j.janxdis.2014.02.004

[37] Chignon JM, Cortes MJ, Martin P, Chabannes JP. Attempted suicide and alcohol dependence: results of an epidemiologic survey. Encephale 1998; 24(4): 347–54. 9809240

[38] Fergusson DM, McLeod GF, Horwood LJ. Childhood sexual abuse and adult developmental outcomes: findings from a 30-year longitudinal study in New Zealand. Child Abuse Negl 2013; 37(9): 664–74. 23623446 10.1016/j.chiabu.2013.03.013

[39] Windle M. Characteristics of alcoholics who attempted suicide: co-occurring disorders and personality differences with a sample of male Vietnam era veterans. J Stud Alcohol 1994; 55(5): 571–7. 7990467 10.15288/jsa.1994.55.571

[40] Conner KR, Ilgen MA. Substance use disorders and suicidal behavior: a conceptual model. Int Handbook Suicide Prev 2016: 110–23.

[41] Murphy GE, Wetzel RD. The lifetime risk of suicide in alcoholism. Arch Gen Psychiatry 1990; 47(4): 383–92. 2181963 10.1001/archpsyc.1990.01810160083012

[42] Sher L. Alcohol and suicide: neurobiological and clinical aspects. ScientificWorldJournal 2006; 6: 700–6. 16799741 10.1100/tsw.2006.146 PMC5917128

[43] López-Goñi JJ, Fernández-Montalvo J, Arteaga A, Haro B. Suicidal attempts among patients with substance use disorders who present with suicidal ideation. Addict Behav 2019; 89: 5–9. 30237111 10.1016/j.addbeh.2018.09.006

[44] Thomasson R, Craig V, Guthrie E. Self-disembowelment during delirium tremens: why early diagnosis is vital. BMJ Case Rep 2016; 24(10): 2016–217258. 10.1136/bcr-2016-217258 PMC5093829 27797817

[45] Charan SH, Reddy CMPK. Genital self mutilation in alcohol withdrawal state complicated with delirium. Indian J Psychol Med 2011; 33(2): 188–90. 22345848 10.4103/0253-7176.92045 PMC3271498

[46] Pompili M, Gibiino S, Innamorati M, Serafini G, Del Casale A, De Risio L, Prolactin and thyroid hormone levels are associated with suicide attempts in psychiatric patients. Psychiatry Res 2012; 200(2): 389–94. 22748186 10.1016/j.psychres.2012.05.010

[47] Seo D, Patrick CJ, Kennealy PJ. Role of serotonin and dopamine system interactions in the neurobiology of impulsive aggression and its comorbidity with other clinical disorders. Aggress Violent Behav 2008; 13(5): 383–95. 19802333 10.1016/j.avb.2008.06.003 PMC2612120

